# Distribution of *Candidatus* Liberibacter species in Eastern Africa, and the First Report of *Candidatus* Liberibacter asiaticus in Kenya

**DOI:** 10.1038/s41598-020-60712-0

**Published:** 2020-03-03

**Authors:** Inusa J. Ajene, Fathiya M. Khamis, Barbara van Asch, Gerhard Pietersen, Nurhussen Seid, Ivan Rwomushana, Fidelis L. O. Ombura, George Momanyi, Pole Finyange, Brenda A. Rasowo, Chrysantus M. Tanga, Samira Mohammed, Sunday Ekesi

**Affiliations:** 1 International Center of Insect Physiology and Ecology, Nairobi, Kenya; 2 Department of Genetics, Stellenbosch University, Stellenbosch, South Africa; 3 Department of Crop Protection, Faculty of Agriculture Ahmadu Bello University, Zaria, Nigeria; 4 CAB International, Nairobi, Kenya; 5 Awassa University, Awassa, Ethiopia; 6 Kenya Plant Health Inspectorate Service, Nairobi, Kenya; 7 Kenya Agricultural and Livestock Research Organization, Matuga, Kenya

**Keywords:** DNA sequencing, Ecology

## Abstract

Huanglongbing (HLB) is a serious disease of *Citrus* sp. worldwide. In Africa and the Mascarene Islands, a similar disease is known as African citrus greening (ACG) and is associated with the bacterium *Candidatus* Liberibacter africanus (Laf). In recent years, *Candidatus* Liberibacter asiaticus (Las) associated with the severe HLB has been reported in Ethiopia. Thus, we aimed to identify the Liberibacter species affecting citrus, the associated vectors in Eastern Africa and their ecological distribution. We assessed the presence of generic Liberibacter in symptomatic leaf samples by quantitative PCR. Subsequently, we sequenced the 50 S ribosomal protein L10 (*rplJ*) gene region in samples positive for Liberibacters and identified the species by comparison with public sequence data using phylogenetic reconstruction and genetic distances. We detected generic Liberibacter in 26%, 21% and 66% of plants tested from Uganda, Ethiopia and Kenya, respectively. The *rplJ* sequences revealed the most prevalent Liberibacters in Uganda and Ethiopia were LafCl (22%) and Las (17%), respectively. We detected Las in Kenya for the first time from three sites in the coastal region. Finally, we modelled the potential habitat suitability of Las in Eastern Africa using MaxEnt. The projection showed large areas of suitability for the pathogen in the three countries surveyed. Moreover, the potential distribution in Eastern Africa covered important citrus-producing parts of Ethiopia, Kenya, Uganda and Tanzania, and included regions where the disease has not been reported. These findings will guide in the development of an integrated pest management strategy to ACG/HLB management in Africa.

## Introduction

Huanglongbing (HLB) is presently one of the most destructive plant diseases affecting citrus groves worldwide^1^. The disease is associated with *Candidatus* Liberibacter asiaticus (Las) and *Candidatus* Liberibacter americanus (Lam), which are phloem-limited, fastidious, gram-negative bacteria belonging to the alpha subdivision of Proteobacteria^2,3^. Las is heat-tolerant and associated with the severe HLB which is transmitted by the Asian citrus psyllid *Diaphorina citri* Kuwayama (Liviidae)^4^*. Diaphorina citri* is distributed in Asia, the United States, Central America, Ethiopia and Brazil^5–10^. In addition to Las and Lam, the citrus-infecting Liberibacter genus contains another species: *Candidatus* Liberibacter africanus (Laf)^1^. Laf is heat-sensitive and is associated with African citrus greening disease (ACG)^11–13^. This pathogen is principally transmitted by *Trioza erytreae* (Del Guercio) (Triozidae), also known as the African citrus triozid^14,15^. Additionally, several subspecies of Laf have been reported, including *Candidatus* Liberibacter subsp. capensis (LafC), *Candidatus* Liberibacter africanus subsp. clausenae (LafCl), *Candidatus* Liberibacter africanus subsp. teclae (LafT), *Candidatus* Liberibacter africanus subsp. vepridis (LafV), and *Candidatus* Liberibacter africanus subsp. zanthoxyli (LafZ)^13,16,17^.

Citrus is the most extensively cultivated fruit crop in Kenya^18,19^ with annual production ranging from 61 to 135 tons between 2012 and 2016^20^. Citrus production provides a source of income for farmers, as well as a ready source of vitamin for home-consumption^19^. However, production has been on the decline in East Africa, particularly in the highland regions^19,21^. Furthermore, fruit yield at the smallholder level is about 10 t/ha, far below the potential of 75 t/ha^18,22^. Several biotic and abiotic factors have been responsible for the decreasing yields, with pests and diseases among the most important^23^. Among the diseases, ACG remains the most prevalent, particularly in the mid to high altitude areas of Eastern Africa and is primarily attributed to Laf^24–26^.

However, in the last decade, Las was reported for the first time in Ethiopia^10^ and more recently in Uganda in 2016^25^, although the reported occurrences in Uganda were subsequently shown to have been misidentifications^27^. That notwithstanding, the confirmed presence of Las in at least one country in Eastern Africa represents a real threat to citrus production, as Las is more heat-tolerant and adapted to the various altitudinal ranges’ characteristic of the countries in this region. Furthermore, recent studies have established that *D. citri* is now established in Kenya and Tanzania^26,28^. This could potentially have even more impact than envisaged as *D. citri* possesses a superior ability as a Las vector^15,29^. It is likely that with the increasing regional trade, similar climatic patterns and the presence of reservoir hosts plants, Las could be more widespread than is currently known and its interactions with Laf and related subspecies is still largely unknown. Current models on the potential spread of HLB are based on suitable climate conditions for the psyllid vector *D. citri*^30,31^. However, the risk of the establishment of HLB is not based solely on the distribution of the vector, because other factors such as environmental requirements may differ between the psyllid and the bacterial pathogen^15,32^. Thus, it becomes therefore necessary to confirm the status and distribution of Liberibacter species and the subspecies in the region. Therefore, this study aimed to asses the status of HLB in three Eastern Africa countries (Ethiopia, Kenya and Uganda) by identifying the associated Liberibacter species in citrus plants and their psyllid vectors, establishing their occurrence, and determining the potential spread to other citrus-producing parts of the region.

## Results

### Presence of citrus greening disease and psyllid vectors

**Figure 1:**
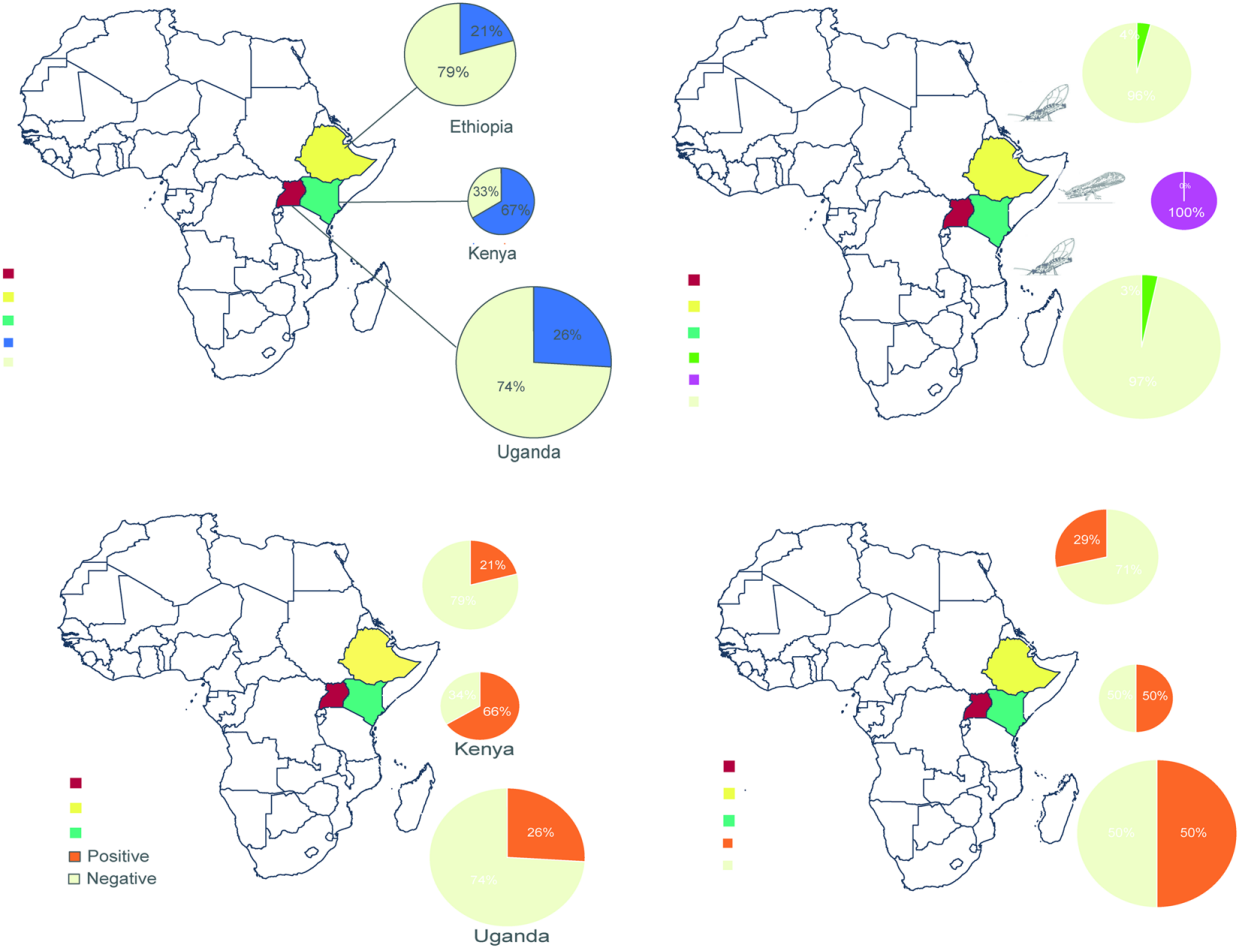
Presence of citrus greening disease symptoms, *Candidatus* Liberibacter species and psyllid vectors, from the survey of citrus in Uganda, Ethiopia and Kenya. (**a**) Proportion of sites where symptomatic trees were found, (**b**) Proportion of sites where the insect vectors *Trioza erytreae* and *Diaphorina citri* were found, (**c**) Generic Liberibacter detected in plants by qPCR, and (**d**) Generic Liberibacters detected in vectors by qPCR. The size of the pie charts is proportional to the total number of sites surveyed.

Fields were monitored in Uganda (300 sites), Ethiopia (170 sites) and Kenya (9 sites). Symptoms typical of citrus greening disease were encountered at study sites in all three countries surveyed and included yellowing, galls and vein degeneration of leaf veins, while dieback was observed in trees with severe disease progression (see Supplementary Fig. S1). Infected trees which were subsequently shown to be Laf-infected showed milder yellowing on the leaves and in most cases, infected leaves were observed on one side of the tree. In cases where Las was detected, the leaf symptoms were more severe and encompassed the entire tree. The survey covered altitudes ranging from lowlands (<1000 m.a.s.l), midlands (1000–1500 m.a.s.l) to highlands (>1500 m.a.s.l.) (see Supplementary Table S1). In Ethiopia, HLB symptoms were not seen at low altitudes (<1000 m; Oromia region) but were found at high altitudes (1,876–2,116 m; Gondar region). Greening disease symptoms were found in 26% of the Ugandan sites, 20.6% of the Ethiopian sites and 66.6% of the sites surveyed in Kenya. (Fig. 1a). *Trioza erytreae* was found at 10 sites in Uganda and seven sites in Ethiopia, but not in any of the sites in Kenya. Conversely, *D. citri* was found at all sites in Kenya at the time of the survey, but not in Uganda or Ethiopia (Fig. 1b).

### Detection of generic Liberibacter by qPCR

The presence of generic Liberibacter in the plant and insect samples collected in Uganda, Ethiopia and Kenya was assessed by qPCR. All samples had the same melting peak as the positive control (see Supplementary Fig. S2). Generic Liberibacter was detected in 78 plant samples collected in Uganda (26%), 35 plant samples in Ethiopia (21%), and six plant samples in Kenya (66%) (Fig. 1c). Liberibacter was also detected in adult *T. erytreae* collected in Uganda (five sites) and Ethiopia (five sites), and from adult *D. citri* collected in Kenya (three sites) (Fig. 1d).

### Identification of Liberibacter species using Sanger sequencing

**Figure 2:**
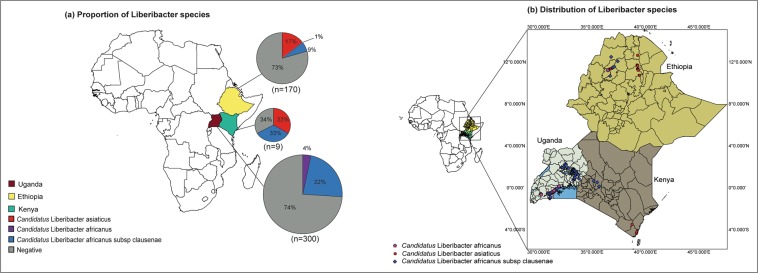
Distribution of *Candidatus* Liberibacter species in Uganda, Ethiopia and Kenya. (**a**) Proportion of *Candidatus* Liberibacter species identified from each country. (**b**) Distribution map showing the sampling sites positive for each *Candidatus* Liberibacter species. The size of the pie charts is proportional to the total number of sites surveyed.

PCR amplifications consistently resulted in the expected product size (approx. 650 bp) from all samples positive for generic Liberibacter in the qPCR assays. BLASTn analyses showed high homology of the new sequences with publicly available Las, Laf and LafCl sequences. In Uganda, 66 sites (22%) were positive for LafCl, while 12 sites (4%) were positive for Laf (Fig. 2a). The sequences showed high homology (up to 99% identity) with publicly available LafCl and Laf sequences. In Ethiopia, 29 sites (17%) were positive for Las, 15 sites (9%) for LafCl, and one site (1%) for Laf (Fig. 2a), and the sequences showed high homology with publicly available Las, Laf and LafCl (all 100%) sequences. In Kenya, three sites (33%) were positive for Las and three sites (33%) for LafCl. The sequences showed high homology with publicly available sequences for Las (100%) and LafCl (99%). The spatial distribution of the species showed that Laf and LafCl occurred together in the western region in Uganda, with LafCl the only species in the eastern region (Fig. 2b). In Ethiopia, LafCl (75%), Las (25%) and Laf (4%) occurred in the Gonder region while only Las was found in Tigray and Wollo regions. In Kenya, Las was present at the Coastal region, while LafCl was found in the western region.

### Identification of Liberibacter species found in Uganda, Ethiopia and Kenya

**Figure 3:**
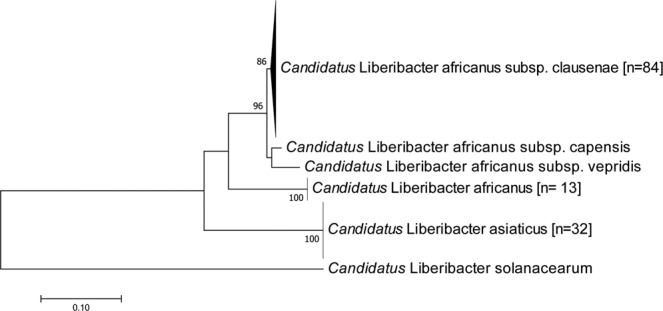
Maximum-Likelihood tree based on a 650 bp alignment of 129 *Candidatus* Liberibacter sequences of the 50 S ribosomal protein L10 (*rplJ)* gene from symptomatic citrus samples collected from Ethiopia (n = 45), Kenya (n = 6) and Uganda (n = 78), and other Liberibacter sequences available in GenBank (n = 6), with *Candidatus* Liberibacter solanacearum as an outgroup. The number of Liberibacter sequences obtained in this study is indicated in square brackets. Branchsupport was based on 1,000 bootstrap replicates.

A Maximum Likelihood tree was built using the sequences obtained in this study combined with representative sequences available in GenBank to assess the phylogenetic and phylogeographic structure of the *rplJ* sequences (Fig. 3). The tree topology indicated that all new sequences were identical to either Las, Laf or LafCl, and no other known Liberibacter species were found. The new sequences from Ethiopia clustered in three distinct clades: one clade with Las, one clade of Laf, and one clade of LafCl. The sequences from Kenya clustered in two clades: one clade with Las, and another formed by LafCl. The new sequences from Uganda clustered in two clades: one clade including publicly available Laf sequences, and another formed by LafCl (Fig. 3). Separate trees were constructed for each country (see Supplementary Fig. S3).

**Figure 4:**
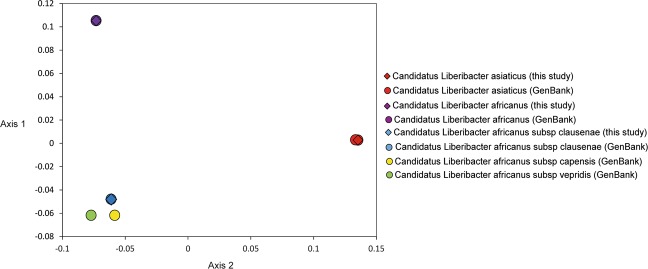
Principal Coordinate Analysis (PCoA) plot representing the genetic distances among Liberibacter sequences (n = 129) from Ethiopia, Kenya, Uganda, and publicly available sequences available in GenBank (n = 144) computed using the classic multidimensional scaling function ‘cmdscale’ in R version 3.5.1.

Genetic distance analyses for the Liberibacter species identification included 129 new sequences and 144 publicly available sequences. For Laf and LafCl, no difference was found between the new sequences and the sequences available on GenBank, as only one haplotype was found for each species (see Supplementary Table S2). All new Las sequences were identical to one of the four haplotypes previously found for Las (see Supplementary Fig. S4). Interspecific genetic distances showed that Las/Laf was the most diverged pair (23.23%) (Table 1). Las was similarly diverged from LafV (22.32%), LafC (20.51%) and LafCl (20.33%). Laf had slightly lower distances from LafCl (15.43%), LafC (16.88%) and LafV (16.88%). The lowest divergences were found between the pairs LafC/LafV (4.36%), LafCl/LafV (3.81%) and LafCl/LafC (2.72%). The PCoA plot based on genetic distances among all Liberibacter showed overlap of the new and publicly available Laf, LafCl and Las sequences, and evidenced the divergence of between Las, Laf, and the cluster comprising LafCl, LafC and LafV (Fig. 4).

**Table 1:** Interspecific mean uncorrected p-distances (%) for *Candidatus* Liberibacter species and subspecies: Las - *Candidatus* Liberibacter asiaticus, Laf - *Candidatus* Liberibacter africanus, LafCl - *Candidatus* Liberibacter africanus subsp. clausenae, LafC - *Candidatus* Liberibacter africanus subsp. capensis and LafV - *Candidatus* Liberibacter africanus subsp. vepridis. Distances were calculated based on a 649 bp alignment of 273 new and publicly available sequences. Standard error estimates are shown above the diagonal. * - Sequences from this study combined with publicly available sequences.

	Laf*	LafC (GenBank)	LafCl*	LafV (GenBank)	Las*
Laf*	—	0.016	0.015	0.015	0.017
LafC (GenBank)	16.88	—	0.007	0.008	0.017
LafCl*	15.43	2.72	—	0.008	0.017
LafV (GenBank)	16.88	4.36	3.81	—	0.018
Las*	23.23	20.51	20.33	22.32	—

### Potential distribution of Las in Africa

**Figure 5:**
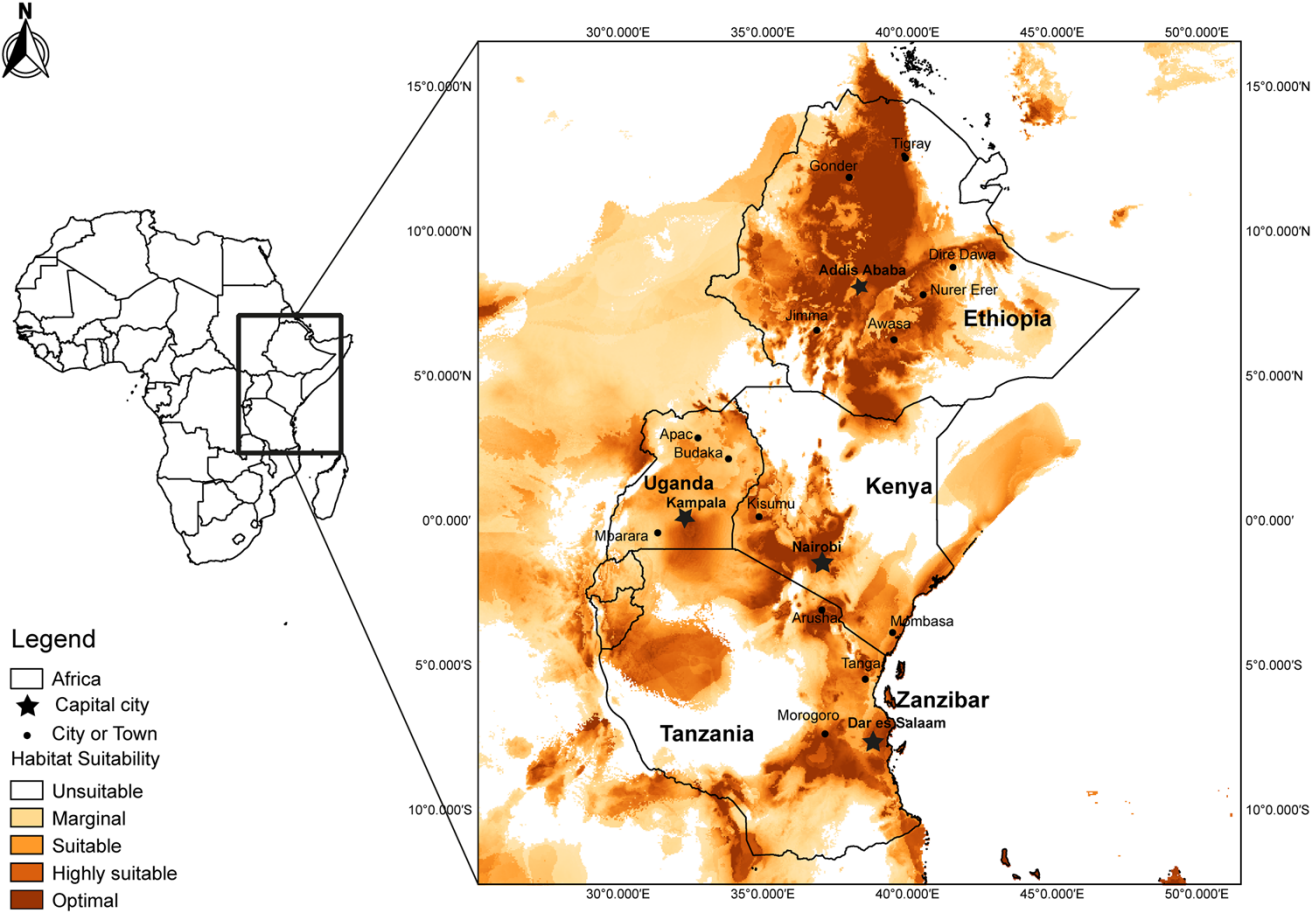
Potential distribution of the Huanglongbing associated with *Candidatus* Liberibacter asiaticus in Eastern Africa, as predicted from the current occurrence locations using global 50-year climate data with MaxEnt.

The modelling of the potential distribution of Las resulted in an AUC value for the current distribution of 0.76125, and the distribution of *D. citri* resulted in an AUC value of 0.84332, indicating a good fit of the prediction in relation to the datasets used. The consensus model showed a potential distribution for HLB covering most of the citrus-producing areas of Eastern Africa. Large areas of optimum potential distribution of HLB were predicted in Ethiopia including Tigray, Gonder, Dire Dawa, Awasa and Jimma, whereas most parts of Uganda showed marginal suitability. The predicted distribution of HLB in Kenya covered the coastal, western and central regions including the areas surveyed (Lunga-Lunga, Matuga and Awasi), while large areas of northern Kenya were predicted to be unsuitable. Large areas of Tanzania showed marginal to optimal suitability for HLB including Arusha, Morogoro, Tanga and Dar es Salaam. The islands of Zanzibar also showed high suitability for this pathogen (Fig. 5).

## Discussion

In this study, we assessed the distribution of Liberibacter species in three Eastern African countries, as well as determined the potential spread of Las in the region. Mild to severe symptoms of citrus greening disease were encountered in different citrus growing agro-ecologies across a range of altitudes from low to high altitude regions in Ethiopia, Kenya and Uganda. Furthermore, HLB symptoms in the Ethiopian highlands appeared to be severe, in contrast to reports that they are less pronounced and disappear above 1,500 m^14^. These symptoms were observed in the absence of the vector *D. citri*. In Uganda, symptomatic plants in the Eastern region presented severe symptoms akin to the HLB infected plants observed in Ethiopia, but we identified the pathogen as LafCl. Citrus plants in the western region, which were infected by Laf, had milder symptoms typical of ACGD.

*Trioza erytreae* adults were found on citrus trees in Ethiopia and Uganda, while *D. citri* adults were found in Kenya. In the three countries, both insect species tested positive for generic Liberibacter. Previous studies have postulated the probable transmission of Las by *T*. erytreae^1,33–35^. Furthermore, previous studies experimentally determined that *T. erytreae* and *D. citri* could both transmit ACG (associated with Laf) and HLB (associated with Las)^36,37^, although *D. citri* was the more effective vector for HLB. The detection of Las in field populations of *T. erytreae* in a previous study^38^ suggests that this species has the potential for also transmitting HLB, particularly to citrus groves at the mid to higher altitude areas of Eastern Africa. Rwomushana *et al*.^28^ have also reported a niche overlap between *D. citri* and *T. erytreae*, which is likely to result in *T. erytreae* picking up HLB from lower-lying areas and potentially spreading it alongside ACG at the mid-altitude areas whose conditions are suitable for this disease. We as yet have no evidence if HLB and ACG can co-exist on the same citrus plant, and we propose further studies to understand the epidemiology of citrus greening in the midst of both pathogens. The risk of spread of HLB to Tanzania also remains high due to the recent invasion of *D. citri* in the country^26^.

The detection of low concentrations of Liberibacter in asymptomatic plants has been problematic even with sensitive diagnostic methods^39^. In Florida, for example, the detection of the pathogen from psyllid vectors is commonly reported long before the detection from the plants^39^. Although the quantity of the Las detected in citrus material was generally low in the Kenyan samples, the Liberibacter was unambiguously identified in plants and vectors.

Phylogenetic relationships and genetic divergence within and between Liberibacter groups were assessed to assist in species identification, as these methods have proven useful provided that the groups under consideration are well represented in the reference dataset^40–42^. Publicly available sequences identified as Las, Laf, LafCl, LafC and LafV provided references for the identification of the new sequences. Our sequences formed a monophyletic cluster with sequences publicly available Las, Laf and LafCl.

No intraspecific divergence was found in Laf or LafCl, as the new sequences were identical to previously reported sequences. For Las, the publicly available sequences comprised four haplotypes, with the most frequent haplotype found in the Americas and Asia. The remaining haplotypes were unique to Oman, India and China, respectively. The new Las sequences were identical to the most frequently occurring haplotype. The intraspecific genetic divergence of Las was less than 0.07%, thus supporting correct species identification of all public and new Las sequences. Interestingly, interspecific comparisons showed low genetic divergence between LafCl, LafC and LafV (average = 3.63%). These Liberibacter are currently classified as subspecies of Laf and were recovered as a group separated from Laf in the ML analysis. Additionally, the average genetic divergence between Laf and the cluster LafCl/LafC/LafV (16.40%) was only slightly lower than the average between Las and the cluster LafCl/LafC/LafV (21.59%). This suggests that the sequences LafCl, LafC and LafV could be different haplotypes representing the same species. Therefore, further analyses will be necessary to establish the species status of this genetic group. Future whole-genome comparisons among Liberibacter will contribute to clarify the relationships among Laf and the haplotypes currently classified as its subspecies.

We identified both Laf and LafCl in Uganda; the two Liberibacter previously found to be widely distributed in the citrus-growing areas in the western and central regions of the country^27^. In our study, LafCl was dominant in the eastern region of the country where Las had been previously misreported^25^. Our results support the hypothesis that the previous record of Las in Uganda was probably a misidentification^27^ therefore, the presence of Las in Uganda remains unconfirmed. On the contrary, our survey in Ethiopia showed that, in addition to LafCl, Las was also widely distributed in the citrus-growing areas of the Amhara region. Our survey in Kenya covered relatively fewer sites than in the other countries; however, we found Las both in citrus plants and in the insect vector *D. citri* in the coastal region. In the western and central parts of the country, we only identified LafCl, as previously reported^43^. Overall, our survey confirms that Las is present in Kenya and that the distribution of LafCl in Uganda and Las in Ethiopia is much more widespread than previously described^10,27^.

Vector-based modelling has been the usual practice for establishing the distribution of HLB. However, a consensus model of the vector and pathogen will give a more robust view of the potential distribution of the disease. Environmental and climate data, including monthly temperature and rain, are assumed to reflect the climate suitability for the growth and development of different organisms, including plant pathogens^44^ (Hijmans, Cameron, Parra, Jones & Jarvis, 2005). Ecological factors such as; temperature, light and water availability, soil fertility, methane and CO_2_ concentration can have varying effects disease development, as pathogens show different responses to these factors^45^. Precipitation and soil moisture have a crucial effect on plant disease establishment, because, most plant diseases are favored by conditions of rain and high air humidity^45^. Temperature is also a key factor in the development of plant diseases, has a significant effect on vector-borne pathogens like Las, Laf and Banana bunchy top virus (BBTV) transmitted by *Pentalonia nigronervosa* and influences the incidence and severity by affecting the vector^46^.

The ensemble approach to HLB distribution carried out in this study indicated large areas of optimum habitat suitability for the pathogen in Ethiopia, including the areas where the disease was detected in our study, as well as the areas where it has previously been reported^10,32^. It also showed areas of habitat suitability in other citrus-producing areas of the country such as Nura-Hera, Awasa, Jimma and Dire Dawa where the disease has not yet been detected. The model showed marginal suitability for Las in eastern Uganda, the major citrus-producing region of the country. Large areas of Tanzania showed marginal to optimal suitability for the pathogen, and the regions where *D. citri* has been detected^28^ were highly suitable. Likewise, the island of Zanzibar, where *D. citri* is present, showed high suitability for the pathogen. The coastal and western regions of Kenya, where citrus production is high, showed optimum suitability. The previous detection of *D. citri* in these regions^28^ and, the subsequent detection of Las in our survey suggests an increased risk of spread of the disease to the citrus producing parts of the central region of Kenya. Kobori *et al*.^47^ reported that adult *D. citri* hardly move after finding a suitable host, they can potentially disperse for at least 2 km within 12 days^48^. Further efforts by phytosanitary authorities are therefore recommended towards preventing further spread of *D. citri* to suitable areas in neighbouring Uganda where this vector was absent.

The spread of Las depends, not only on climate suitability, but also on the presence/absence of the vectors (*D. citri* and *T. erytreae*)^27^. Additionally, human-mediated activities have been implicated in the long-distance transmission of HLB^39,49,50^. Therefore, phytosanitary and quarantine measures need to be implemented particularly in countries where the pathogen is not yet present and included in integrated management strategies. Considering the potential ability of *T. erytreae* to transmit Las, the monitoring and detection of the vector using molecular techniques is now of utmost importance for successful management. An integrated strategy involving the systematic destruction of infected trees and effective psyllid control should be deployed to stem the spread of the disease.

Despite the increasing knowledge on the complex of bacteria causing citrus greening disease in Ethiopia, Kenya and Uganda, the genetic diversity of the vector populations in both countries remain unknown. Some citrus psyllid populations may have inherently differential ability to transmit citrus greening disease, and certain types and strains of Liberibacter may be inherently more transmissible than others^6^. Therefore, research to unravel the population genetic structure of the citrus psyllids in different agro-ecological zones of the region needs to be carried out to inform the integrated pest management approaches for citrus greening disease in Africa.

The HLB threat to the citrus industry in Africa is clearly increasing. This study showed that the Las pathogen associated with HLB is widespread in Ethiopia and present in other regions of the country far dispersed from previously surveyed areas. We also report the presence of Las in Kenya for the first time, suggesting that there is an increased risk for the geographical spread of HLB further afar on the continent. Our modelling of the potential distribution of HLB highlights the high risk of disease spread to areas previously deemed unsuitable. We found Las thriving at higher -altitude regions with cooler temperatures in Ethiopia and the warm climate regions of Kenya; therefore, the spread of HLB is presently more evident. Our study highlighted the dominance of Laf distribution in Uganda and the potential spread of HLB to other citrus-producing parts of East Africa. We recommend that urgent measures need to be put in place to halt the further spread of the disease in other neighbouring areas, including strict phytosanitary measures particularly for citrus seedlings.

## Methods

### Sample collection and DNA extraction

Field surveys were carried out for ACG, HLB and their associated insect vectors (*T. erytreae* and *D. citri*) in citrus orchards and backyard gardens in Uganda (300 sites), Ethiopia (170 sites) and Kenya (9 sites), from March 2017 to December 2018. The citrus species sampled included sweet orange (*Citrus sinensis* L. Osbeck.), lemon (*Citrus limon* L. Osbeck) and tangerine (*Citrus reticulata* Blanco) Leaf samples were collected from plants that exhibited the typical citrus greening symptoms; defoliation, mottling, twig and tree dieback, and lopsided fruit in the most severe cases. Infected leaf samples (four leaves per tree) were individually collected into plastic bags, and stored at 4 °C. Psyllids encountered on infected trees were also aspirated from the trees and stored in vials with 96% ethanol for later DNA extraction. At each site where psyllids were found, 20 specimens per site were collected for screening which bacterium was present. Geographic positioning coordinates (GPS) of the sampling sites were obtained using a Garmin eTrex20 instrument (GARMIN, USA) and recorded for each site.

In the laboratory, individual psyllids and plant leaves were surface-sterilised by completely submerging the insect/leaf in a petri dish containing 3% sodium hypochlorite for three seconds and rinsing thrice with distilled water. Total DNA from individual petioles was extracted using the Isolate II Plant DNA Kit (Bioline, London, UK), while total DNA from individual psyllids was extracted using the Isolate II Genomic DNA Kit (Bioline) then eluted to a final volume of 50 μl. DNA extracts were checked for purity and concentration using a Nanodrop 2000/2000c Spectrophotometer (Thermo Fischer Scientific, Wilmington, USA). DNA extracts within the A_260 nm_/A_280nm_ ratio range of 1.8 to 2.0 were stored at −20 °C then used in downstream processes.

### Quantitative PCR assay for detection of generic Liberibacter

Quantitative PCR (qPCR) was used to screen for the presence of generic Liberibacter in each plant and psyllid DNA extract. A 1160 bp region of a mitochondrial gene (16 s rRNA) conserved in Liberibacter was targeted using the forward primer LibUF^16,51^ and the reverse primer HLBr^9^ (see supplementary Table S3). Reactions were prepared in a final volume of 10 µl containing 0.5 pmol µl^−1^ of each primer, 2x Maxima SYBR Green/ROX qPCR Master Mix (Thermo Fischer Scientific, Wilmington, USA), and 15 ng µl^−1^ of DNA template. The assays were performed in a Stratagene MX3005P qPCR instrument (Agilent Technologies, California, USA), under the following conditions: an initial denaturation step of 10 min at 95 °C, 40 cycles of 95 °C for 30 s, 61 °C for 45 s and 72 °C for 1 min, followed by one dissociation cycle of 95 °C for 1 min, 55 °C for 30 s and 95 °C for 30 s. All assays were performed using three replicates and a negative control. Fluorescence was measured, and quantification cycle (Ct) values were determined using MxPro–Mx3005 P Software (Agilent Technologies). The specificity and purity of the amplicons were determined by the dissociation curve. A threshold of Ct < 31 was used as the cut-off value for the detection of generic Liberibacter as the HLB primers, are reliable at Ct < 30 while at Ct > 30 they are unreliable in differentiating whether the Liberibacter is present^52^. Ct values were recorded for all the reactions. The amplification curves for Ct determination and the melting curves for temperature estimations at the peak of the curves were analysed using the MxPro – Mx 3005P qPCR software.

### PCR amplification and Sanger sequencing for species identification of Liberibacters

Species identification was done on all samples positive for generic Liberibacter in the qPCR assays. A 650 bp region of the 50 S ribosomal protein L10 (*rplJ*) gene was used in conventional PCR for the identification of the Liberibacter species Las, Laf, LafCl, LafZ, LafC and LafZ. The fragment was amplified using primer pair A2 and J5 (see supplementary Table S3)^53^. Reactions were carried out in a total volume of 20 μl containing 5X My Taq reaction buffer (5 mM dNTPs, 15 mM MgCl_2_, stabilizer and enhancer) (Bioline), 0.5 pmol µl^−1^ of each primer, 0.5 mM MgCl_2_ (Thermo Fischer Scientific, Wilmington, USA), 0.0625 U µl^−1^ MyTaq DNA polymerase (Bioline), and 15 ng µl^−1^ of DNA template. PCR was run in a Mastercycler Nexus gradient thermal cycler (Eppendorf, Germany), using the following conditions: initial denaturation for 2 min at 95 °C, followed by 40 cycles of denaturation for 30 s at 95 °C, annealing for 45 s at an optimized annealing temperature of 58.7 °C and extension for 1 min at 72 °C, and a final extension step of 10 min at 72 °C. PCR products were separated in a 1.5% agarose gel stained with 10 mg ml^−1^ ethidium bromide (Sigma-Aldrich, Gmbh, Germany). Gel bands were analysed and documented using KETA GL imaging system trans-illuminator (Wealtec Corp, Meadowvale Way Sparks, Nevada, USA). PCR products were purified using Isolate II PCR and Gel Kit (Bioline) following the manufacturer’s instructions. The purified PCR products were bi-directionally sequenced by Macrogen Inc Europe Laboratory, The Netherlands, using the forward and reverse PCR primers. Forward and reverse sequences for each sample were trimmed to remove primer regions and assembled to obtain a consensus using BioEdit v7.2.5^54^. All new sequences were deposited in GenBank (accession numbers MK542517–MK542519).

### Liberibacter sequence analyses

Sequence identity was determined for all sequences using Basic Local Alignment Search Tool (BLAST). Multiple sequence alignments for phylogenetic reconstruction and estimates of pairwise genetic distances were performed using MAFFT^55^ in Geneious Prime version 2019.0.4. (https://www.geneious.com).

Phylogenetic reconstruction for the representation of the patterns of evolutionary divergence included all sequences generated in this study and 144 additional sequences of Liberibacter species available in GenBank (see Supplementary Table S4), with *Candidatus* Liberibacter solanacearum as an outgroup. Public sequences shorter than 650 bp, containing nucleotide ambiguities, and non-overlapping with the *rplJ* region were excluded from downstream analyses. To avoid excessively dense trees in the genetic clustering analyses, duplicate haplotypes in the public sequences were identified and deleted using Geneious, and only one sequence per species was used as a representative. Maximum-likelihood (ML) trees were reconstructed using the Tamura-Nei model^56^ implemented in MEGA X^57^. Branch support was estimated by 1,000 bootstrap replicates.

Intra and interspecific sequence divergence were calculated using the uncorrected p-distance model in MEGA X. Standard errors were obtained using 1,000 bootstrap replicates. All the new and publicly available sequences were used, and duplicate sequences were not removed. Multidimensional scaling analysis was carried out using the ‘cmdscale’ function in R software version 3.5.1^58^ on the genetic distance matrix to generate the plot for principal coordinate axis (PCoA). The R code is publicly available at https://github.com/InusaJacob/Principal-Coordinate-axes.

### Predicted distribution HLB in Eastern Africa

The potential distribution of Las and the high-risk areas for HLB in Eastern Africa were predicted using the Maximum Entropy (MaxEnt) model^59^. Both the bacteria and its vector (*D. citri*) were modelled individually and the resulting risk maps were overlaid to get a consensus map of the potential distribution of HLB in Eastern Africa. In addition to the results of the current survey, reports of HLB and *D. citri* occurrence were obtained from the CABI Invasive Species Compendium^60^. All points positive for Las from the current study areas were excluded from the presence points and added to the background points used in the model to avoid sampling bias. Environmental predictors used for the modelling of Las and *D. citri* included 19 bioclimatic variables for the current baseline average (1950–2000) acquired from the WorldClim database (http://www.worldclim.org/). For each model, presence locations (occurrence data) were compared against background points to avoid model overfitting of spatially clustered presence points, and inability to predict spatially independent data. We used 75% of the presence data to train the model, and 25% for model validation. The models were run with 5,000 iterations and >10,000 background points. The Area Under the Receiver Operator Curve (AUROC) was used to validate the performance of each model. AUC values above 0.5 connote sites with high predicted suitability. To obtain a consensus of the two models, we rescaled the raster outputs from the individual models to uniform values between 0 and 1. We combined the individual models weighted by their AUC scores, subtracted 0.5 and squared the results to give further weight to the higher AUC values, thus obtaing the weighted average. Finally, we made a Raster Stack of our individual model predictions and computed the average. All models were run in Maxent version 3.3.3k, and the data was exported as ASCII files for enhanced visualization with QGIS software version 2.18.15^61^.

## Supplementary information

Supplementary Dataset.

## Data Availability

All relevant data are within the paper and supplementary materials.
