# Supplementary material for: Distribution of Candidatus Liberibacter species in Eastern Africa, and the First Report of Candidatus Liberibacter asiaticus in Kenya
Source: Sci Rep. 2020 Mar 3;10:3919. doi: 10.1038/s41598-020-60712-0 (PMC7054587; doi:10.1038/s41598-020-60712-0)
Supplement: Supplementary file 1 — Supplementary Dataset. [file 41598_2020_60712_MOESM1_ESM.docx]

**Distribution of *Candidatus* Liberibacter species in Eastern Africa, and the First Report of *Candidatus* Liberibacter asiaticus in Kenya**

Inusa J. Ajene^1,2,3^, Fathiya M. Khamis^1, *^, Barbara van Asch^2^, Gerhard Pietersen^2^, Nurhussen Seid^5^, Ivan Rwomushana^4^, Fidelis L.O. Ombura^1^_,_ George Momanyi^6^, Pole Finyange^7^, Brenda A. Rasowo^1^, Chrysantus Mbi Tanga^1^, Samira Mohammed^1,^ & Sunday Ekesi^1,^

^1^International Center of Insect Physiology and Ecology, Nairobi, Kenya, ^2^Department of Genetics, Stellenbosch University, Stellenbosch, South Africa, ^3^Department of Crop Protection, Faculty of Agriculture Ahmadu Bello University, Zaria, Nigeria, ^4^CAB International, Nairobi, Kenya, ^5^Awassa University, Awassa, Ethiopia, Kenya Plant Health Inspectorate Service Nairobi, Kenya^6^, Kenya Agricultural and Livestock Research Organization, Matuga, Kenya^7^.

*Corresponding author, email: [fkhamis@icipe.org](mailto:fkhamis@icipe.org)

**Supplementary Materials**


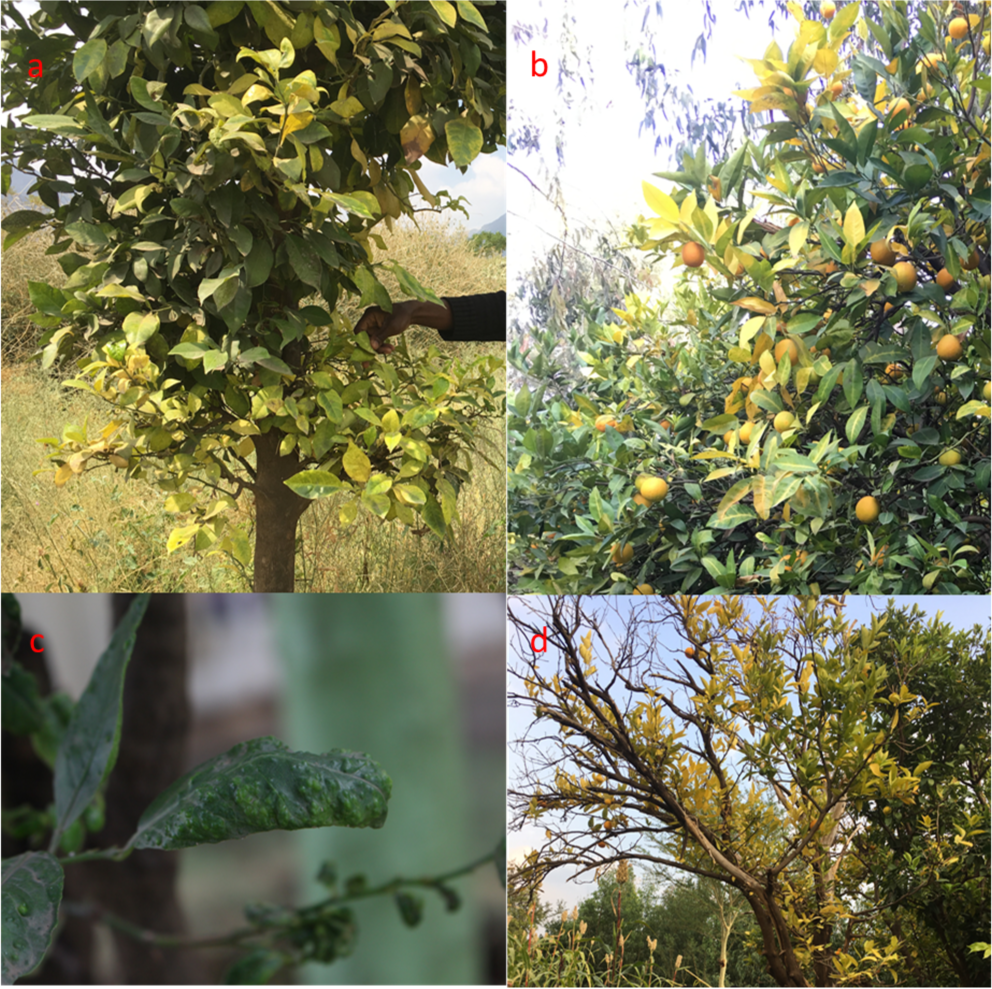


**Supplementary Figure S1.** Symptoms typical of Huanglongbing disease found in citrus plants in Ethiopia. a) Severe yellowing of leaves, b) Lopsided fruit and yellowing of leaves, c) Leaf galls caused by *T*rioza *erytreae* nymph feeding, and d) Defoliation, yellowing and dieback on an orange tree.


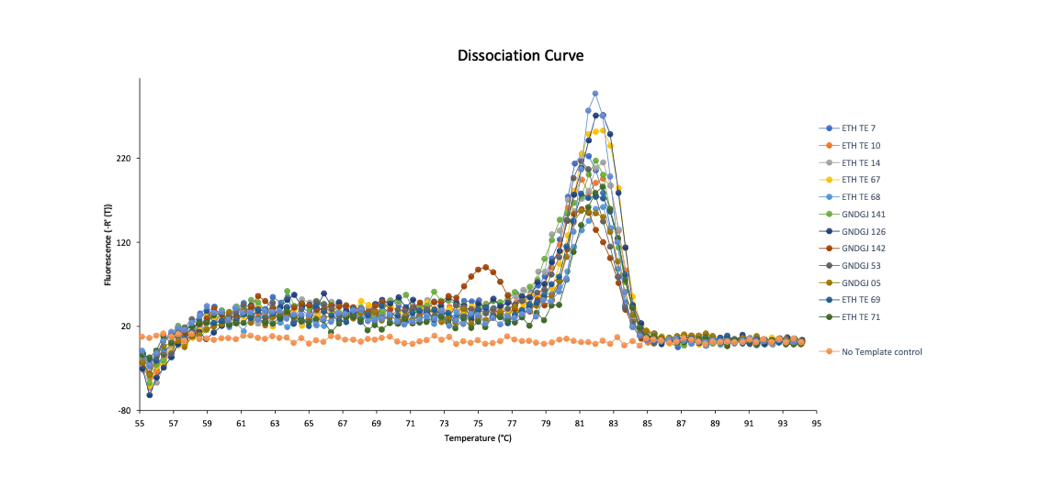
**Supplementary Figure S2.** Melting curve for Liberibacter 16S ribosomal DNA gene in representative plant and insect vector samples. ETH TE - Liberibacter samples from *Trioza erytreae* vector, GNDGJ . Liberibacter samples from citrus plants.


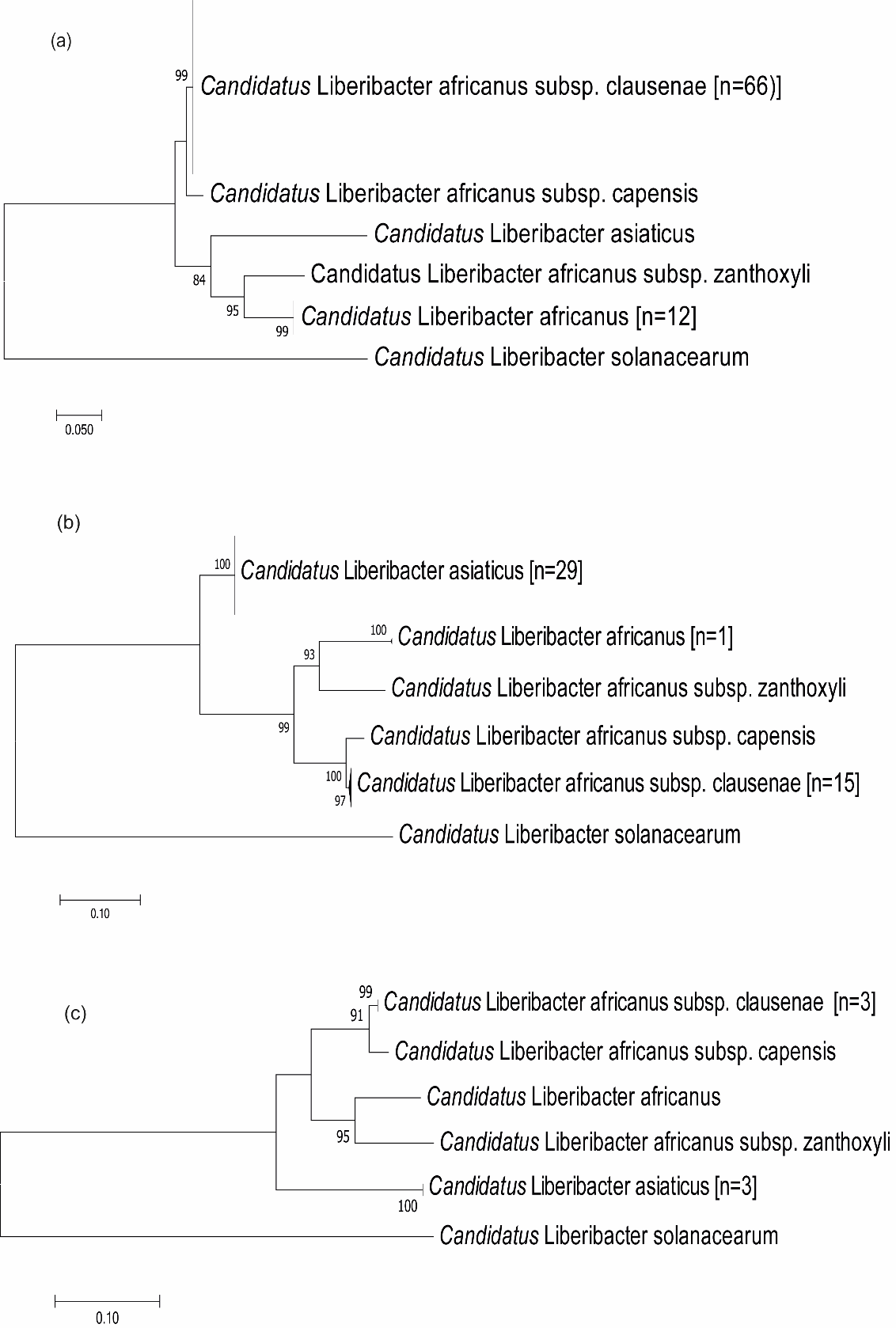


**Supplementary Figure S3.** Maximum-likelihood trees based on a 650 bp alignment of 129 sequences of the 50S ribosomal protein L10 (*rplJ)* gene of Liberibacter found on symptomatic citrus samples collected from (a) Ethiopia (n = 45), (b) Kenya (n = 6), and (c) Uganda (n = 78), and publicly available representative Liberibacter sequences with *Candidatus* Liberibacter solanacearum as an outgroup. The number of Liberibacter sequences obtained in this study is indicated in square brackets. Bootstrap values were based on 1,000 replicates.


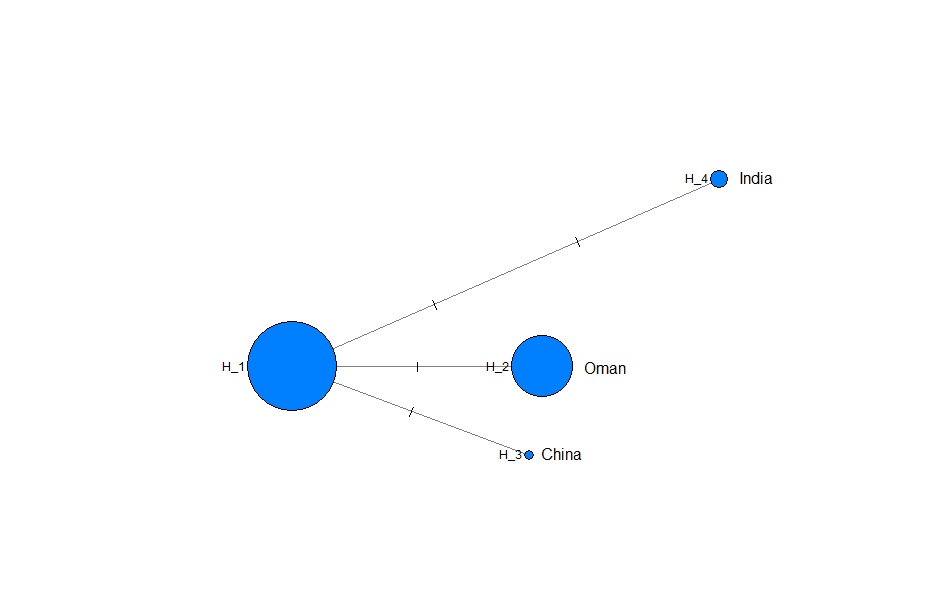


**Supplementary Figure S4.** Haplotype network representing the relationships among the new and publicly available *Candidatus* Liberibacter asiaticus sequences used in this study. The size of the circles is proportional to the number of sequences in each haplotype.

**Supplementary Table S1.** Sampling locations where *Candidatus* Liberibacter species were identified from citrus plants by *rplJ* gene in Uganda, Ethiopia and Kenya. Las: *Candidatus* Liberibacter asiaticus*,* Laf: *Candidatus* Liberibacter africanus, Laf-cl: *Candidatus* Liberibacter subspecies Clausenae

| **Country** | **Region** | **Location** | **Altitude (m.a.s.l)** | **Citrus system** | **Liberibacter Identity (*rplJ*)** | **Vector Found** |
| --- | --- | --- | --- | --- | --- | --- |
| Uganda | Western | Naro Zardi | 1457 | Research Orchard | Laf-cl | *T. erytreae* |
|  |  | Katojo | 1439 | Backyard garden | Laf-cl | *T. erytreae* |
|  |  | Kyarwabugunda | 1419 | Backyard garden | Laf-cl | *T. erytreae* |
|  |  | Ruharo | 1415 | Small scale rural farm | Laf | *T. erytreae* |
|  | Central | Kyabigondo | 1259 | Backyard garden | Laf-cl | *T. erytreae* |
|  |  | Lyatonde3 | 1332 | Backyard garden | Laf-cl | *T. erytreae* |
|  |  | Kyanasanjya | 1228 | Backyard garden | Laf-cl | *T. erytreae* |
|  |  | kijjasi | 1258 | Backyard garden | Laf-cl | *T. erytreae* |
|  |  | Mukusamvu | 1175 | Backyard garden | Laf-cl | *T. erytreae* |
|  |  | Lukoma | 1189 | Backyard garden | Laf-cl | *T. erytreae* |
|  |  | Kawuto | 1164 | Small scale rural farm | Laf-cl | *T. erytreae* |
|  |  | Namabare | 1262 | Small scale rural farm | Laf-cl | *T. erytreae* |
|  |  | Makondo | 1279 | Backyard garden | Laf-cl | *T. erytreae* |
|  |  | Kiswera | 1262 | Backyard garden | Laf-cl | *T. erytreae* |
|  |  | Kaswa | 1292 | Backyard garden | Laf-cl | *T. erytreae* |
|  |  | Bajja3 | 1180 | Backyard garden | Laf-cl | *T. erytreae* |
|  |  | Buganga | 1168 | Backyard garden | Laf-cl | *T. erytreae* |
|  |  | Buganga2 | 1143 | Small scale rural farm | Laf-cl | *T. erytreae* |
|  |  | Bubule3 | 1167 | Backyard garden | Laf-cl | *T. erytreae* |
|  |  | Kyengeza | 1278 | Backyard garden | Laf | *T. erytreae* |
|  |  | Kaswa | 1260 | Backyard garden | Laf | *T. erytreae* |
|  |  | Kyamenyamingo | 1288 | Backyard garden | Laf | *T. erytreae* |
|  |  | Kasana | 1279 | Backyard garden | Laf | *T. erytreae* |
|  |  | Bajja | 1180 | Backyard garden | Laf | *T. erytreae* |
|  |  | Bubule | 1167 | Backyard garden | Laf | *T. erytreae* |
|  |  | Bugonzi | 1216 | Backyard garden | Laf | *T. erytreae* |
|  | Eastern | Teibu | 1056 | Small scale rural farm | Laf-cl | *T. erytreae* |
|  |  | Akere | 1058 | Small scale rural farm | Laf-cl | *T. erytreae* |
|  |  | Akere 2 | 1056 | Backyard garden | Laf-cl | *T. erytreae* |
|  |  | Hospital Atik | 1051 | Small scale rural farm | Laf-cl | *T. erytreae* |
|  |  | Atik | 1040 | Backyard garden | Laf-cl | *T. erytreae* |
|  |  | Agulo | 1038 | Commercial orchard | Laf-cl | *T. erytreae* |
|  |  | Aboko | 1037 | Commercial orchard | Laf-cl | *T. erytreae* |
|  |  | Aduku | 1048 | Commercial orchard | Laf-cl | *T. erytreae* |
|  |  | Akoremo | 1059 | Commercial orchard | Laf-cl | *T. erytreae* |
|  |  | Abedi-woro | 1092 | Commercial orchard | Laf-cl | *T. erytreae* |
|  |  | Ngetta | 1076 | Commercial orchard | Laf-cl | *T. erytreae* |
|  |  | Telela | 1102 | Commercial orchard | Laf-cl | *T. erytreae* |
|  |  | Telela2 | 109 | Commercial orchard | Laf-cl | *T. erytreae* |
|  |  | Aminyango | 108 | Commercial orchard | Laf-cl | *T. erytreae* |
|  |  | Gweng Abara | 1121 | Commercial orchard | Laf-cl | *T. erytreae* |
|  |  | Ocokcan | 1099 | Commercial orchard | Laf-cl | *T. erytreae* |
|  |  | Adidun | 1045 | Commercial orchard | Laf-cl | *T. erytreae* |
|  |  | Akani | 1077 | Commercial orchard | Laf-cl | *T. erytreae* |
|  |  | Awase | 1067 | Commercial orchard | Laf-cl | *T. erytreae* |
|  |  | Serere TC | 1130 | Research orchard | Laf-cl | *T. erytreae* |
|  |  | Oburen | 1108 | Small scale rural farm | Laf-cl | *T. erytreae* |
|  |  | Sapir | 1118 | Small scale rural farm | Laf-cl | *T. erytreae* |
|  |  | Omukunyo | 1094 | Small scale rural farm | Laf-cl | *T. erytreae* |
|  |  | Otaba | 1101 | Small scale rural farm | Laf-cl | *T. erytreae* |
|  |  | Otete | 1098 | Commercial orchard | Laf-cl | *T. erytreae* |
|  |  | Arabaka | 1092 | Commercial orchard | Laf-cl | *T. erytreae* |
|  |  | Busitema Uni | 1122 | Research orchard | Laf-cl | *T. erytreae* |
|  |  | Arapai Uni | 1102 | Research orchard | Laf-cl | *T. erytreae* |
|  |  | Opero | 1103 | Commercial orchard | Laf-cl | *T. erytreae* |
|  |  | Alake | 1087 | Commercial orchard | Laf-cl | *T. erytreae* |
|  |  | Awoja | 1049 | Commercial orchard | Laf-cl | *T. erytreae* |
|  |  | Kapir | 1061 | Commercial orchard | Laf-cl | *T. erytreae* |
|  |  | Olupe | 1106 | Commercial orchard | Laf-cl | *T. erytreae* |
|  |  | Okutai | 1081 | Commercial orchard | Laf-cl | *T. erytreae* |
|  |  | Obokora | 1103 | Commercial orchard | Laf-cl | *T. erytreae* |
|  |  | Aoloko | 1132 | Commercial orchard | Laf-cl | *T. erytreae* |
|  |  | Kapokina | 1113 | Commercial orchard | Laf-cl | *T. erytreae* |
|  |  | Bukedea | 1133 | Commercial orchard | Laf-cl | *T. erytreae* |
|  |  | Kachumbala | 1142 | Commercial orchard | Laf-cl | *T. erytreae* |
|  |  | Butuwongore | 1082 | Commercial orchard | Laf-cl | *T. erytreae* |
|  |  | Namalemba | 1103 | Commercial orchard | Laf-cl | *T. erytreae* |
|  |  | Nakawunje | 1115 | Commercial orchard | Laf-cl | *T. erytreae* |
|  |  | Namutumba | 1119 | Commercial orchard | Laf-cl | *T. erytreae* |
|  |  | Buyange | 1085 | Commercial orchard | Laf-cl | *T. erytreae* |
|  |  | Butamba | 1075 | Commercial orchard | Laf-cl | *T. erytreae* |
|  |  | Mazuba | 1067 | Commercial orchard | Laf-cl | *T. erytreae* |
|  |  | Kibuku | 1081 | Commercial orchard | Laf-cl | *T. erytreae* |
|  |  | Nakatende | 1107 | Commercial orchard | Laf-cl | *T. erytreae* |
|  |  | Lambo | 1159 | Commercial orchard | Laf-cl | *T. erytreae* |
|  |  | Molo1 | 1193 | Commercial orchard | Laf-cl | *T. erytreae* |
| Ethiopia | Amhara | Dangila | 2122 | Small scale rural farm | Laf | *T. erytreae* |
|  |  | Dangila2 | 2123 | Small scale rural farm | Laf-cl | *T. erytreae* |
|  |  | Dangila3 | 2116 | Backyard garden | Las | *T. erytreae* |
|  |  | Shuwabere | 1978 | Small scale rural farm | Las | *T. erytreae* |
|  |  | Abchbele-mariam | 1970 | Small scale rural farm | Las | *T. erytreae* |
|  |  | Insude | 1959 | Small scale rural farm | Las | *T. erytreae* |
|  |  | Insude2 | 1964 | Small scale rural farm | Las | *T. erytreae* |
|  |  | Insude3 | 1992 | Small scale rural farm | Las | *T. erytreae* |
|  |  | Insude4 | 2005 | Small scale rural farm | Las | *T. erytreae* |
|  |  | Achabere | 1982 | Backyard garden | Laf-cl | *T. erytreae* |
|  |  | Achabere2 | 1958 | Backyard garden | Laf-cl | *T. erytreae* |
|  |  | Matafa duda | 1936 | Backyard garden | Laf-cl | *T. erytreae* |
|  |  | Addis Zemen | 1920 | Backyard garden | Laf-cl | *T. erytreae* |
|  |  | Addis Zemen2 | 1923 | Backyard garden | Laf-cl | *T. erytreae* |
|  |  | Bohona | 1922 | Backyard garden | Laf-cl | *T. erytreae* |
|  |  | Zenzelima | 1867 | Backyard garden | Las | *T. erytreae* |
|  |  | Zenzelima2 | 1876 | Backyard garden | Laf-cl | *T. erytreae* |
|  |  | Sasaberete | 1891 | Backyard garden | Las | *T. erytreae* |
|  |  | Bure | 2460 | Small scale rural farm | Laf-cl | *T. erytreae* |
|  |  | Bureau of Agric | 2021 | Commercial orchard | Laf-cl | *T. erytreae* |
|  | Wollo | Mola gerado | 1953 | Small scale rural farm | Las | - |
|  |  | Goshuha | 1880 | Backyard garden | Las | - |
|  |  | Gola | 1935 | Backyard garden | Las | - |
|  |  | Weira Amba | 1891 | Commercial orchard | Las | - |
|  |  | Mersa | 161 | Backyard garden | Las | - |
|  |  | Mersa2 | 1679 | Backyard garden | Las | - |
|  |  | Mersa3 | 1592 | Backyard garden | Las | - |
|  |  | Ambasel | 1725 | Backyard garden | Las | - |
|  |  | Jare | 1685 | Backyard garden | Las | - |
|  |  | Pasomile | 1805 | Backyard garden | Las | - |
|  |  | Hayk | 2002 | Backyard garden | Las | - |
|  |  | Hayk2 | 2029 | Backyard garden | Las | - |
|  |  | Abuabu | 2113 | Backyard garden | Las | - |
|  |  | Milamile | 1436 | Small scale rural farm | Las | - |
|  | Tigray | Adishewi | 1657 | Backyard garden | Las | - |
| Kenya | Coast | Lunga-Lunga | 50 | Backyard garden | Las | *D. citri* |
|  |  | Matuga | 55 | Research orchard/ Nursery | Las | *D. citri* |
|  |  | Muhaka | 55 | Research orchard | Las | *D. citri* |
|  | Western | Awasi | 1344 | Backyard garden | Laf-cl | *D. citri* |
|  |  | Soin | 1543 | Backyard garden | Laf-cl | *D. citri* |
|  |  | Koitamburot | 1409 | Backyard garden | Laf-cl | *D. citri* |

**Supplementary Table S2.** Intraspecific mean uncorrected p-distances (%) for citrus greening associated *Candidatus* Liberibacter species: based on a 649 bp alignment of 273 new and publicly available 50S ribosomal protein L10 gene region sequences. Standard error estimates (SE) are shown in the last column. Las - *Candidatus* Liberibacter asiaticus, Laf - *Candidatus* Liberibacter africanus, LafCl - *Candidatus* Liberibacter africanus subsp. clausenae, LafC - *Candidatus* Liberibacter africanus subsp. capensis and LafV - *Candidatus* Liberibacter africanus subsp. vepridis

|  | Number of sequences | Number of haplotypes | Intraspecific p-distance (mean, %) | SE |
| --- | --- | --- | --- | --- |
| LafCl (this study) | 84 | 1 | 0.00 | 0.000 |
| LafCl (GenBank) | 3 | 1 | 0.00 | 0.000 |
| LafC (GenBank) | 4 | 1 | 0.00 | 0.000 |
| Laf (GenBank) | 14 | 1 | 0.00 | 0.000 |
| Laf (this study) | 13 | 1 | 0.00 | 0.000 |
| Las (GenBank) | 123 | 4 | 0.10 | 0.001 |
| Las (this study) | 32 | 1 | 0.00 | 0.000 |

**Supplementary Table S3.** Primers used in the qPCR assay for the detection of generic Liberibacter, and PCR amplification and bidirectional sequencing of the 50S ribosomal protein L10 region in *Candidatus* Liberibacter species (**in bold**) for species and sub-species identification. Liberibacter DNA was obtained from citrus plants, and the insect vectors *Trioza erytreae* and *Diaphorina citri* in Uganda, Ethiopia and Kenya.

| Primer | Assay | Primer sequence (5’-3’) | PCR product (bp) |
| --- | --- | --- | --- |
| LibUF | qPCR | GGCAGGCCTAACACATGC | ˜1160 |
| HLBr |  | 5’-GCGTTATCCCGTAGAAAAAGGTAG | ˜1160 |
| **A2** | **PCR** | **TATAAAGGTTGACCTTTCGAGTTT** | **˜650** |
| **J5** |  | **ACAAAAGCAGAAATAGCACGAACAA** | **˜650** |

**Supplementary Table S4.** List of publicly available and new ribosomal protein L10 (rplJ) sequences (excluding sequences: shorter than 650 bp and containing nucleotide ambiguities) used in the phylogenetic analyses of *Candidatus* Liberibacter species recovered from citrus plants in Uganda, Ethiopia and Kenya, with GenBank accession numbers. Sequences generated in this study are shown in **bold**.

| **Organism** | **Accession** | **Country (region)** |
| --- | --- | --- |
| ***Candidatus* Liberibacter africanus** | **MK542518.1** | **Uganda** |
| *Candidatus* Liberibacter africanus | GU120033.1 | South Africa |
| *Candidatus* Liberibacter africanus | GU120041.1 | South Africa |
| *Candidatus* Liberibacter africanus | GU120034.1 | South Africa |
| *Candidatus* Liberibacter africanus | GU120037.1 | South Africa |
| *Candidatus* Liberibacter africanus | GU120038.1 | South Africa |
| *Candidatus* Liberibacter africanus | GU120043.1 | South Africa |
| *Candidatus* Liberibacter africanus | GU120032.1 | South Africa |
| *Candidatus* Liberibacter africanus | GU120042.1 | South Africa |
| *Candidatus* Liberibacter africanus | GU120040.1 | South Africa |
| *Candidatus* Liberibacter africanus | GU120036.1 | South Africa |
| *Candidatus* Liberibacter africanus | GU120044.1 | South Africa |
| *Candidatus* Liberibacter africanus | GU120039.1 | South Africa |
| *Candidatus* Liberibacter africanus | GU120035.1 | South Africa |
| *Candidatus* Liberibacter africanus subsp. capensis | JF419553.1 | South Africa |
| *Candidatus* Liberibacter africanus subsp. capensis | JF419554.1 | South Africa |
| *Candidatus* Liberibacter africanus subsp. capensis | JF419555.1 | South Africa |
| *Candidatus* Liberibacter africanus subsp. capensis | KJ197225.1 | South Africa |
| *Candidatus* Liberibacter africanus subsp. clausenae | KX770999.1 | Tanzania |
| *Candidatus* Liberibacter africanus subsp. clausenae | KX770998.1 | Uganda |
| ***Candidatus* Liberibacter africanus subsp. clausenae** | **MK542519.1** | **Uganda** |
| *Candidatus* Liberibacter africanus subsp. clausenae | KJ189106.1 | South Africa |
| *Candidatus* Liberibacter africanus subsp. vepridis | KJ189105.1 | South Africa |
| *Candidatus* Liberibacter asiaticus | JX430435.1 | USA (California) |
| *Candidatus* Liberibacter asiaticus | KC133065.1 | China (Guangxi) |
| *Candidatus* Liberibacter asiaticus | KC133067.1 | China (Hubei) |
| *Candidatus* Liberibacter asiaticus | KC133068.1 | China (Hubei) |
| *Candidatus* Liberibacter asiaticus | KR919749.1 | USA (Texas) |
| *Candidatus* Liberibacter asiaticus | MF769715.1 | India (Assam) |
| *Candidatus* Liberibacter asiaticus | MF769714.1 | India (Assam) |
| *Candidatus* Liberibacter asiaticus | KM889670.1 | India |
| *Candidatus* Liberibacter asiaticus | KM889671.1 | India |
| *Candidatus* Liberibacter asiaticus | KT164846.1 | India |
| *Candidatus* Liberibacter asiaticus | JF346109.1 | Bhutan |
| *Candidatus* Liberibacter asiaticus | MG418841.1 | Venezuela |
| *Candidatus* Liberibacter asiaticus | FJ394022.1 | Cuba |
| *Candidatus* Liberibacter asiaticus | GQ890155.1 | Ethiopia |
| *Candidatus* Liberibacter asiaticus | GQ890156.1 | Ethiopia |
| *Candidatus* Liberibacter asiaticus | JN211014.1 | China |
| *Candidatus* Liberibacter asiaticus | MK542517.1 | Ethiopia |
| ***Candidatus* Liberibacter asiaticus** | **MK542517.1** | **Ethiopia** |
| *Candidatus* Liberibacter asiaticus | JN211015.1 | China |
| *Candidatus* Liberibacter asiaticus | JN211016.1 | China |
| *Candidatus* Liberibacter asiaticus | JN211017.1 | China |
| *Candidatus* Liberibacter asiaticus | JN211018.1 | China |
| *Candidatus* Liberibacter asiaticus | JN211019.1 | China |
| *Candidatus* Liberibacter asiaticus | MF767288.1 | India (West Bengal) |
| *Candidatus* Liberibacter asiaticus | MF694639.1 | India (West Bengal) |
| *Candidatus* Liberibacter asiaticus | KF699074.1 | French West Indies |
| *Candidatus* Liberibacter asiaticus | KF699075.1 | French West Indies |
| *Candidatus* Liberibacter asiaticus | KF699076.1 | French West Indies |
| *Candidatus* Liberibacter asiaticus | KF699081.1 | French West Indies |
| *Candidatus* Liberibacter asiaticus | KF699077.1 | French West Indies |
| *Candidatus* Liberibacter asiaticus | KF699088.1 | French West Indies |
| *Candidatus* Liberibacter asiaticus | KF699078.1 | French West Indies |
| *Candidatus* Liberibacter asiaticus | KF699080.1 | French West Indies |
| *Candidatus* Liberibacter asiaticus | KF699082.1 | French West Indies |
| *Candidatus* Liberibacter asiaticus | KF699089.1 | French West Indies |
| *Candidatus* Liberibacter asiaticus | KF699090.1 | French West Indies |
| *Candidatus* Liberibacter asiaticus | KF699083.1 | French West Indies |
| *Candidatus* Liberibacter asiaticus | KF699091.1 | French West Indies |
| *Candidatus* Liberibacter asiaticus | KF699084.1 | French West Indies |
| *Candidatus* Liberibacter asiaticus | KF699085.1 | French West Indies |
| *Candidatus* Liberibacter asiaticus | KF699092.1 | French West Indies |
| *Candidatus* Liberibacter asiaticus | KF699086.1 | French West Indies |
| *Candidatus* Liberibacter asiaticus | KF699079.1 | French West Indies |
| *Candidatus* Liberibacter asiaticus | KF699087.1 | French West Indies |
| *Candidatus* Liberibacter asiaticus | MF769717.1 | India (Maharashtra) |
| *Candidatus* Liberibacter asiaticus | KT164840.1 | India |
| *Candidatus* Liberibacter asiaticus | KT164841.1 | India |
| *Candidatus* Liberibacter asiaticus | KT164842.1 | India |
| *Candidatus* Liberibacter asiaticus | KT164843.1 | India |
| *Candidatus* Liberibacter asiaticus | KT164844.1 | India |
| *Candidatus* Liberibacter asiaticus | KT164845.1 | India |
| *Candidatus* Liberibacter asiaticus | KC137978.1 | India |
| *Candidatus* Liberibacter asiaticus | JF261098.1 | Iran (Kerman) |
| *Candidatus* Liberibacter asiaticus | GU074017.1 | India (Jampui Hills) |
| *Candidatus* Liberibacter asiaticus | HQ335314.1 | Iran |
| *Candidatus* Liberibacter asiaticus | MF767289.1 | India (Sikkim) |
| *Candidatus* Liberibacter asiaticus | JX284242.1 | India (Meghalaya) |
| *Candidatus* Liberibacter asiaticus | JX284243.1 | India (Meghalaya) |
| *Candidatus* Liberibacter asiaticus | KC137980.1 | India |
| *Candidatus* Liberibacter asiaticus | JX284244.1 | India (Meghalaya) |
| *Candidatus* Liberibacter asiaticus | MG418842.1 | Venezuela |
| *Candidatus* Liberibacter asiaticus | JN211020.1 | China |
| *Candidatus* Liberibacter asiaticus | JN211021.1 | China |
| *Candidatus* Liberibacter asiaticus | KC596024.1 | Iran |
| *Candidatus* Liberibacter asiaticus | LC090236.1 | Indonesia (Bali) |
| *Candidatus* Liberibacter asiaticus | AB859772.1 | Mexico (Colima) |
| *Candidatus* Liberibacter asiaticus | AB859773.1 | Mexico (Sinaloa) |
| *Candidatus* Liberibacter asiaticus | AB859774.1 | Mexico (Nayarit) |
| *Candidatus* Liberibacter asiaticus | DQ303211.1 | China (Guangdong) |
| *Candidatus* Liberibacter asiaticus | KC477386.1 | India (Uttar Pradesh) |
| *Candidatus* Liberibacter asiaticus | JX455746.1 | USA (Los Angeles) |
| *Candidatus* Liberibacter asiaticus | KY990823.1 | Iran |
| *Candidatus* Liberibacter asiaticus | KY990824.1 | Iran |
| *Candidatus* Liberibacter asiaticus | KC477383.1 | India (Karnataka) |
| *Candidatus* Liberibacter asiaticus | KC477380.1 | India (Karnataka) |
| *Candidatus* Liberibacter asiaticus | KC477381.1 | India (Karnataka) |
| *Candidatus* Liberibacter asiaticus | KC477384.1 | India (Karnataka) |
| *Candidatus* Liberibacter asiaticus | KY550692.1 | Oman |
| *Candidatus* Liberibacter asiaticus | KY550693.1 | Oman |
| *Candidatus* Liberibacter asiaticus | KY550694.1 | Oman |
| *Candidatus* Liberibacter asiaticus | KC477375.1 | India (Assam) |
| *Candidatus* Liberibacter asiaticus | KC477376.1 | India (Assam) |
| *Candidatus* Liberibacter asiaticus | JQ973890.1 | India |
| *Candidatus* Liberibacter asiaticus | JQ973891.1 | India |
| *Candidatus* Liberibacter asiaticus | JQ973892.1 | India |
| *Candidatus* Liberibacter asiaticus | JQ973894.1 | India |
| *Candidatus* Liberibacter asiaticus | JQ973895.1 | India |
| *Candidatus* Liberibacter asiaticus | KC816565.1 | India (Mizoram) |
| *Candidatus* Liberibacter asiaticus | KY550690.1 | Oman |
| *Candidatus* Liberibacter asiaticus | KY550695.1 | Oman |
| *Candidatus* Liberibacter asiaticus | KY550696.1 | Oman |
| *Candidatus* Liberibacter asiaticus | KY550697.1 | Oman |
| *Candidatus* Liberibacter asiaticus | KY550698.1 | Oman |
| *Candidatus* Liberibacter asiaticus | KY550704.1 | Oman |
| *Candidatus* Liberibacter asiaticus | KY550705.1 | Oman |
| *Candidatus* Liberibacter asiaticus | KY550706.1 | Oman |
| *Candidatus* Liberibacter asiaticus | KY550707.1 | Oman |
| *Candidatus* Liberibacter asiaticus | KY550699.1 | Oman |
| *Candidatus* Liberibacter asiaticus | KY550700.1 | Oman |
| *Candidatus* Liberibacter asiaticus | KY550701.1 | Oman |
| *Candidatus* Liberibacter asiaticus | KY550702.1 | Oman |
| *Candidatus* Liberibacter asiaticus | KY550708.1 | Oman |
| *Candidatus* Liberibacter asiaticus | KY550709.1 | Oman |
| *Candidatus* Liberibacter asiaticus | KY550685.1 | Oman |
| *Candidatus* Liberibacter asiaticus | KY550678.1 | Oman |
| *Candidatus* Liberibacter asiaticus | KY550679.1 | Oman |
| *Candidatus* Liberibacter asiaticus | KY550680.1 | Oman |
| *Candidatus* Liberibacter asiaticus | KY550681.1 | Oman |
| *Candidatus* Liberibacter asiaticus | KY550682.1 | Oman |
| *Candidatus* Liberibacter asiaticus | KY550683.1 | Oman |
| *Candidatus* Liberibacter asiaticus | KY550686.1 | Oman |
| *Candidatus* Liberibacter asiaticus | KY550688.1 | Oman |
| *Candidatus* Liberibacter asiaticus | KY550703.1 | Oman |
| *Candidatus* Liberibacter asiaticus | KY550684.1 | Oman |
| *Candidatus* Liberibacter asiaticus | KY550691.1 | Oman |
| *Candidatus* Liberibacter asiaticus | KY550687.1 | Oman |
| *Candidatus* Liberibacter asiaticus | KY550689.1 | Oman |
| *Candidatus* Liberibacter asiaticus | KY550672.1 | Oman |
| *Candidatus* Liberibacter asiaticus | KY550673.1 | Oman |
| *Candidatus* Liberibacter asiaticus | KY550674.1 | Oman |
| *Candidatus* Liberibacter asiaticus | KY550676.1 | Oman |
| *Candidatus* Liberibacter asiaticus | KY550675.1 | Oman |
| *Candidatus* Liberibacter asiaticus | KY550677.1 | Oman |
| *Candidatus* Liberibacter solanacearum | MF041971.1 | USA (California) |
